# Continuity of care for patients with chronic disease: a registry-based observational study from Norway

**DOI:** 10.1093/fampra/cmab107

**Published:** 2021-09-18

**Authors:** Sahar Pahlavanyali, Øystein Hetlevik, Jesper Blinkenberg, Steinar Hunskaar

**Affiliations:** Department of Global Public Health and Primary Care, University of Bergen, Bergen, Norway; Department of Global Public Health and Primary Care, University of Bergen, Bergen, Norway; Department of Global Public Health and Primary Care, University of Bergen, Bergen, Norway; National Centre for Emergency Primary Health Care, NORCE Norwegian Research Centre, Bergen, Norway; Department of Global Public Health and Primary Care, University of Bergen, Bergen, Norway; National Centre for Emergency Primary Health Care, NORCE Norwegian Research Centre, Bergen, Norway

**Keywords:** chronic disease, continuity of care, general practice, healthcare system, Norway, observational study

## Abstract

**Background:**

Continuity of care (CoC) is accepted as a core value of primary care and is especially appreciated by patients with chronic conditions. Nevertheless, there are few studies investigating CoC for these patients across levels of healthcare.

**Objective:**

This study aims to investigate CoC for patients with somatic chronic diseases, both with regular general practitioners (RGPs) and across care levels.

**Methods:**

We conducted a registry-based observational study by using nationwide consultation data from Norwegian general practices, out-of-hours services, hospital outpatient care, and private specialists with public contracts. Patients with diabetes mellitus (type I or II), asthma, chronic obstructive pulmonary disease, or heart failure in 2012, who had ≥2 consultations with these diagnoses during 2014 were included. CoC was measured during 2014 by using the usual provider of care (UPC) index and Bice–Boxerman continuity of care score (COCI). Both indices have a value between 0 and 1.

**Results:**

Patients with diabetes mellitus comprised the largest study population (*N* = 79,165) and heart failure the smallest (*N* = 4,122). The highest mean UPC and COCI were measured for patients with heart failure, 0.75 and 0.77, respectively. UPC increased gradually with age for all diagnoses, while COCI showed this trend only for asthma. Both indices had higher values in urban areas.

**Conclusions:**

Our findings suggest that CoC in Norwegian healthcare system is achieved for a majority of patients with chronic diseases. Patients with heart failure had the highest continuity with their RGP. Higher CoC was associated with older age and living in urban areas.

**Key Messages**

- Continuity of care is high for majority of patients with chronic diseases.
- High continuity of care is associated with old age.
- Continuity with regular general practitioner is lower in rural areas.

## Background

Continuity of care (CoC) refers to the coherent and connected care experienced by a patient over time, in harmony with patient’s health requirements and personal situations.^1,2^ CoC is regarded as a core value of primary care and indicates a dimension of quality of care in general practice.^3^ Evidence suggests that CoC in primary care leads to decreased risk of emergency visits and hospital admissions,^4–8^ reduced usage of healthcare and its costs,^9–12^ increased utilization of preventive care,^13^ and lower mortality risk.^2,14^ CoC with specialist physicians is also associated with reduced mortality.^15,16^ Thus, CoC is not only relevant for primary care providers but is valued by all providers at all levels of healthcare, indicating that CoC may still be achieved for patients who are mainly treated in specialist care.

Benefits are also observed from the patients’ perspectives. Patients appreciate seeing the same doctor,^17^ especially patients with chronic conditions or heavy healthcare use seem to value continuity.^18^

In Norway, most patients with chronic conditions are treated by their regular general practitioner (RGP) and referred to specialist healthcare if necessary. The Norwegian patient list system introduced in 2001 guarantees a RGP for every resident,^19^ and was designed with a goal to initiate CoC with RGP. A study from 2009 supports this accomplishment by concluding that 78% of consultations were with RGPs.^20^ Previous Norwegian studies focus on continuity with RGPs and have demonstrated that CoC is associated with decreased hospital readmissions^21^ and reduced utilization of specialist care.^22,23^ Despite the known benefits of CoC, we have scarce evidence on CoC for patients with chronic diseases in Norway, a group of patients anticipated to especially benefit from CoC.

The aim of this study was to determine whether CoC is achieved among RGPs, and across care levels for patients with certain chronic diseases: asthma, chronic obstructive pulmonary disease (COPD), diabetes mellitus, and heart failure. Additionally, we investigated CoC in relation to patient characteristics, such as age, rural/urban residence, and educational level.

## Methods

We conducted an observational registry-based study by linking healthcare and population data from several national registries, from the years 2012 and 2014.

### Data sources

Claims data from Norwegian general practices and out-of-hours (OOH) services were obtained from Control and Payment of Reimbursement to Health Service Providers database (KUHR), which is managed by The Norwegian Health Economics Administration (HELFO). HELFO also administers GP registry, which supplied data on each patient’s RGP. The International Classification of Primary Care, second edition (ICPC-2) is used to code diagnoses in primary healthcare.

Norwegian Patient Registry (NPR) provided data for patient contacts with private specialists with public contracts (PSPCs) and hospital outpatient clinics. Contact data from Bergen (Norway’s second largest city) OOH service during office hours, were also received from NPR, as such data were not included in KUHR. The International Statistical Classification of Diseases and Related Health Problems version 10 (ICD-10) is applied for NPR diagnoses.

Statistics Norway (SSB) supplied data on centrality (urban/rural) classes^24^ and patients’ educational level.^25^

Data from registries were linked by SSB using each patient’s national identification number. SSB anonymized data for research by replacing this number by a project specific identification number. A detailed description of Norwegian healthcare system and registries is published elsewhere.^26^

### Study population

All patients in Norway with at least 1 consultation in primary healthcare during 2012 with a diagnosis code for asthma, COPD, diabetes mellitus type I, diabetes mellitus type II, or heart failure were identified.

Consultations include visits to patient’s RGP or other GPs, home visits by RGPs or other GPs, patient visits to OOH services, and home visits by OOH services physician. In the Norwegian patient list system, more than 99% of residents are registered with a RGP.^27^ Nevertheless, patients may consult other GPs when the RGP is not available. Since around 95% of GPs work in group practices,^28^ seeing *other GPs* implies most often to GPs in the same practice as the patient’s RGP. However, the GPs’ practice affiliation cannot be identified in the available data.

We registered consultations regarding these diagnoses from general practices, OOH services, PSPCs, and hospital outpatient clinics, throughout 2014. Consultations from NPR database also contained codes for relevant symptoms for each chronic condition.

We included patients with at least 2 consultations with appropriate codes during 2014 in the study populations. Patients with more than 1 chronic disease were included in all relevant populations. Some ICPC codes for diabetes type I or II were used interchangeably between 2012 and 2014. Therefore, we included patients with either of these 2 diagnoses in 1 population.

Patients who had changed their RGP or were not registered on RGP scheme list during 2014 were excluded. We also excluded those who had migrated out of Norway or died by 31.12.2014.

All RGPs, GPs, PSPCs, and OOH service physicians were regarded as individual providers in our analyses, while physicians at each hospital outpatient clinic or Bergen OOH service during office hours were counted as 1 provider per clinic.

### Measures of CoC

We calculated CoC by using 2 continuity indices, which are frequently used for measuring CoC based on claims data.^4^ The *usual provider of care (UPC) index* is calculated as proportion of numbers of consultations (*n*) by a specific provider (RGP in this study) divided by total number of consultations (*N*) over a defined period; UPC= *n*/*N*.^29^*Bice–Boxerman continuity of care* score (*COCI*) is an index reliant to both number of providers and number of consultations with each provider. This index measures the degree to which patients visit several providers by counting total number of visits (*N*), total number of visits to *i*th provider (*n*_*i*_), and total number of providers (*p*),^5,30^ reflecting total CoC across care levels.


$$\begin{array}{l} COCI=\frac{\sum_{i=1^{n_{i}^{2}}}^{\;p}-N}{N(N-1)}. \end{array}$$

Both indices have a value between 0 and 1, with 1 demonstrating the highest possible continuity and 0 indicating full discontinuity of care. In this study, we define values ≥0.75 as high CoC.^31^

### Covariates

Patients’ age, sex, centrality index, educational level, number of consultations with both RGP and other care providers, and number of providers for each patient during 2014 were determined. Age was categorized into 6 subgroups (≤18, 19–44, 45–54, 55–64, 65–74, and ≥75 years). SSB centrality index assorts municipalities in 6 levels.^24^ In this study, we refer to areas with centrality index 1–4 as urban and 5–6 as rural areas. Educational level was classified in 3 main groups according to the highest fulfilled education: low (elementary school or less), medium (upper secondary school), and high (university and higher education).^25^

### Statistical analyses

Descriptive analyses, including frequency, percentage, mean, and SD for both indices, were carried out for each population.

We performed a log-binomial regression analysis with a generalized linear model for each index to investigate the association between COC and patient characteristics. UPC and COCI were the dependent variables and were both dichotomized into low (<0.75) and high (0.75–1). Patient’s gender, age group, and centrality index were entered as predictors. Relative risk (RR), 95% confidence interval (CI), and *P* values were calculated. The unadjusted model for each independent variable was analyzed separately. We excluded educational level variable from the adjusted model analyses because of many missing values for asthma (31%). Missing values were few for other independent variables.

All analyses were done using Stata 16.1. (StataCorp. 2019. *Stata Statistical Software: Release 16.* College Station, TX: StataCorp LLC.)

## Results

**Fig. 1.**
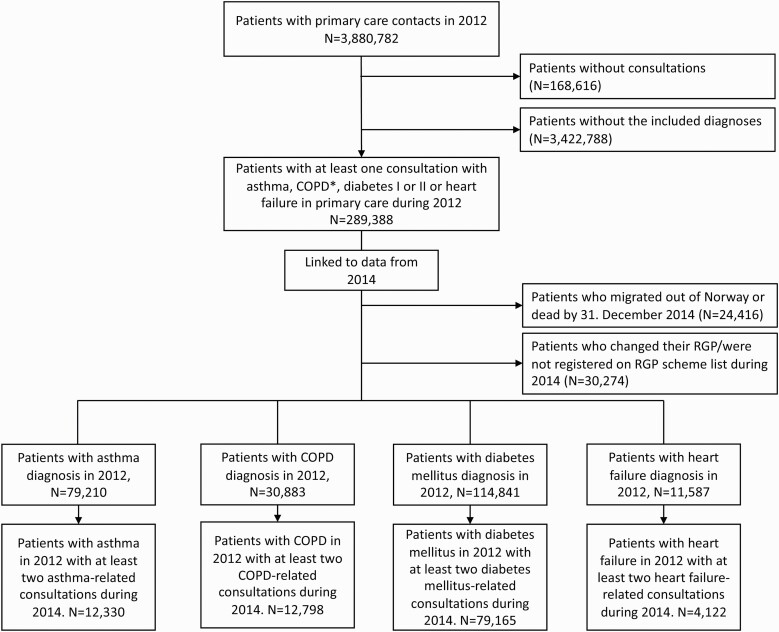
Flow chart showing the inclusion and exclusion process and number of patients available for analysis after being diagnosed in 2012 and having at least 2 disease-related consultations in 2014 for the 4 chosen diseases: asthma, COPD, diabetes mellitus, and heart failure. Patients with more than one of the chronic diseases were included in all relevant study populations. *COPD, chronic obstructive pulmonary disease.

Figure 1 shows inclusion and exclusion process, and how the 4 study populations were defined. Having fulfilled the criterion of at least 2 disease-related consultations in 2014, 12,330 patients with asthma, 12,798 with COPD, 79,165 with diabetes mellitus (type I or II), and 4,122 with heart failure were available for analyses. Each study population was analyzed separately.

Table 1 shows characteristics of patients in each study population. Asthma group had the youngest population, with a third of patients ≤18 years old, a mean age of 40.3, and a decreasing number of patients by increasing age. It also had the lowest mean number of consultations. The high percentage of missing values for educational level in asthma reflects the young age with no obtained educational level.

**Table 1.** Characteristics of the patients in the 4 study populations with diagnoses: asthma, COPD, diabetes mellitus, and heart failure during 2014.

Diagnoses	Asthma		COPD^a^		Diabetes mellitus		Heart failure	
	N	(%)	N	(%)	N	(%)	N	(%)
Total	12,330	100	12,798	100	79,165	100	4,122	100
Gender								
Female	7,108	(57.6)	6,509	(50.9)	34,888	(44.1)	1,865	(45.3)
Male	5,222	(42.4)	6,289	(49.1)	44,277	(55.9)	2,257	(54.7)
Age groups (years)								
Mean age	40.3		70.5		63.9		78.4	
≤18	3,930	(31.9)	11	(0.09)	661	(0.8)	4	(0.1)
19–44	2,200	(17.8)	96	(0.8)	6,650	(8.4)	57	(1.4)
45–54	1,674	(13.6)	580	(4.5)	10,784	(13.6)	119	(2.9)
55–64	1,874	(15.2)	2,453	(19.2)	18,674	(23.6)	366	(8.9)
65–74	1,632	(13.2)	5,207	(40.7)	24,125	(30.5)	787	(19.1)
≥75	1,020	(8.3)	4,451	(34.8)	18,271	(23.1)	2,789	(67.7)
Centrality index^b^								
1 (most urban)	2,526	(20.5)	1,875	(14.7)	13,647	(17.2)	659	(15.9)
2	3,253	(26.4)	3,377	(26.4)	19,327	(24.4)	1,010	(24.5)
3	3,221	(26.1)	3,508	(27.4)	21,546	(27.2)	1,151	(27.9)
4	2,199	(17.8)	2,491	(19.5)	15,446	(19.5)	764	(18.5)
5	864	(7.0)	1,082	(8.5)	7,030	(8.9)	401	(9.7)
6 (most rural)	267	(2.2)	453	(3.5)	2,169	(2.7)	137	(3.3)
Educational level								
Low	2,862	(23.2)	5,831	(45.6)	27,474	(34.7)	1,662	(40.3)
Medium	3,559	(28.9)	5,834	(45.6)	36,888	(46.6)	1,843	(44.7)
High	2,134	(17.3)	1,036	(8.1)	13,362	(16.9)	582	(14.1)
Missing	3,775	(30.7)	97	(0.7)	1,441	(1.8)	35	(0.9)
Consultations in 2014								
25th percentile	2		2		3		3	
50th percentile	3		4		4		4	
Mean	3		5		5		6	
75th percentile	4		6		5		7	

Educational level: low (elementary school or less), medium (upper secondary school), and high (university and higher education).

^a^COPD = chronic obstructive pulmonary disease.

^b^Centrality index 1 represents the most urban and 6 the most rural.

Gender distribution was quite even for COPD, in contrast to asthma with more female patients. Diabetes mellitus and heart failure were both male dominated. For COPD, the mean age was 70.5 years old and a third was ≥75 years, while very few patients were less than 45 years. Less than 1 in 10 of COPD patients had higher education.

Diabetes mellitus comprised the largest population and included patients with both diabetes mellitus type I and type II. Mean age was 63.9 years, with 1 in 10 ≤45 years of age.

Heart failure was the smallest group of patients while having the largest number of consultations. This was also the oldest population with two thirds of patients being ≥75 years.

### UPC index

For RGPs as usual providers the mean UPC for asthma, COPD, diabetes mellitus, and heart failure were 0.59, 0.66, 0.70, and 0.75, respectively (Table 2). There were no significant differences in UPC by gender for patients with COPD or diabetes mellitus, while female patients with asthma and heart failure had higher UPC. UPC increases gradually by increasing age, and the oldest age group has the highest UPC for all 4 diagnoses. Patients ≤18 years old with diabetes mellitus had a very low UPC.

**Table 2.** The UPC index and Bice–Boxerman continuity of care score measured for patients with asthma, COPD, diabetes mellitus, or heart failure during 2014.

Indices	UPC^a^				COCI^b^			
Diagnoses	Asthma	COPD^c^	Diabetes mellitus	Heart failure	Asthma	COPD	Diabetes mellitus	Heart failure
	Mean (SD)	Mean (SD)	Mean (SD)	Mean (SD)	Mean (SD)	Mean (SD)	Mean (SD)	Mean (SD)
Total	0.59 (0.40)	0.66 (0.35)	0.70 (0.34)	0.75 (0.34)	0.65 (0.41)	0.62 (0.38)	0.67 (0.34)	0.77 (0.32)
Gender								
Female	0.61 (0.39)	0.65 (0.35)	0.70 (0.34)	0.79 (0.32)	0.65 (0.41)	0.61 (0.38)	0.67 (0.34)	0.81 (0.30)
Male	0.55 (0.41)	0.66 (0.35)	0.70 (0.34)	0.72 (0.35)	0.66 (0.42)	0.62 (0.38)	0.67 (0.35)	0.74 (0.33)
Age groups (years)								
≤18	0.43 (0.41)	0.57 (0.47)	0.09 (0.17)	0.50 (0.58)	0.60 (0.43)	0.80 (0.36)	0.75 (0.28)	1 (0.00)
19–44	0.58 (0.39)	0.66 (0.37)	0.50 (0.37)	0.59 (0.42)	0.62 (0.42)	0.65 (0.40)	0.58 (0.35)	0.73 (0.36)
45–54	0.64 (0.38)	0.66 (0.36)	0.66 (0.35)	0.61 (0.37)	0.66 (0.39)	0.62 (0.39)	0.64 (0.35)	0.66 (0.35)
55–64	0.69 (0.37)	0.66 (0.35)	0.72 (0.32)	0.69 (0.36)	0.69 (0.39)	0.62 (0.38)	0.67 (0.34)	0.70 (0.33)
65–74	0.70 (0.37)	0.65 (0.35)	0.73 (0.32)	0.70 (0.35)	0.72 (0.38)	0.61 (0.37)	0.68 (0.34)	0.71 (0.34)
≥75	0.74 (0.36)	0.67 (0.35)	0.76 (0.31)	0.79 (0.33)	0.75 (0.38)	0.62 (0.38)	0.72 (0.34)	0.80 (0.30)
Centrality index^d^								
1 (most urban)	0.59 (0.41)	0.67 (0.34)	0.73 (0.33)	0.78 (0.32)	0.67 (0.40)	0.63 (0.37)	0.70 (0.34)	0.80 (0.29)
2	0.60 (0.40)	0.70 (0.34)	0.72 (0.33)	0.77 (0.33)	0.67 (0.40)	0.66 (0.37)	0.69 (0.34)	0.79 (0.30)
3	0.60 (0.40)	0.67 (0.35)	0.71 (0.33)	0.76 (0.34)	0.66 (0.41)	0.63 (0.37)	0.67 (0.34)	0.78 (0.32)
4	0.60 (0.40)	0.66 (0.35)	0.69 (0.34)	0.75 (0.34)	0.64 (0.41)	0.60 (0.38)	0.66 (0.35)	0.76 (0.32)
5	0.49 (0.40)	0.57 (0.37)	0.65 (0.36)	0.69 (0.37)	0.55 (0.43)	0.52 (0.39)	0.62 (0.37)	0.72 (0.36)
6 (most rural)	0.46 (0.41)	0.43 (0.39)	0.56 (0.38)	0.59 (0.39)	0.55 (0.44)	0.47 (0.38)	0.57 (0.38)	0.65 (0.38)
Educational level								
Low	0.65 (0.39)	0.65 (0.35)	0.72 (0.33)	0.77 (0.34)	0.68 (0.40)	0.61 (0.38)	0.68 (0.34)	0.80 (0.31)
Medium	0.66 (0.38)	0.66 (0.35)	0.71 (0.33)	0.76 (0.34)	0.68 (0.40)	0.62 (0.38)	0.67 (0.34)	0.77 (0.32)
High	0.64 (0.38)	0.67 (0.35)	0.67 (0.35)	0.69 (0.35)	0.67 (0.40)	0.63 (0.37)	0.64 (0.35)	0.72 (0.33)

Distributed by patients’ gender, age, centrality index and educational level, and the patient’s RGP as the usual provider. Educational level: low (elementary school or less), medium (upper secondary school), and high (university and higher education).

^a^UPC = usual provider of care index.

^b^COCI = Bice–Boxerman continuity of care score.

^c^COPD = chronic obstructive pulmonary disease.

^d^Centrality index 1 represents the most urban and 6 the most rural.

UPC was higher in urban areas. There were no associations between educational levels and UPC, apart from a slight decrease in UPC from low to high educational levels for diabetes mellitus and heart failure (Table 2).

### COCI index

The lowest mean COCI was measured for patients with COPD (0.62), while patients with heart failure had the highest (0.77) (Table 2). Like UPC, female patients with heart failure had a higher mean than male patients, otherwise there were small differences based on gender.

For asthma, COCI increased gradually by increasing age. In contrast to UPC, the mean COCI for patients ≤18 years old for COPD, diabetes mellitus, and heart failure, had higher values compared with the other age groups. It is important to note, there were only 4 patients ≤18 years old with heart failure and they all achieved full continuity.

Mean COCI was similar for urban areas but decreased in rural ones. It was also higher for patients with heart failure having low educational level compared with those with higher educational level. There were no other significant trends observed for educational level (Table 2).

### Adjusted log-binomial regression models

Table 3 shows shares of patients with UPC ≥0.75 for study populations by gender, age, and centrality index. For asthma, COPD, and diabetes mellitus about half of patients reached this high level of UPC, while for heart failure about two thirds had UPC ≥0.75. Table 3 also demonstrates results from the adjusted log-binomial regression model for UPC index (unadjusted results not shown). Female patients with heart failure had a statistically significant higher RR of high UPC (RR = 1.13), while female patients with COPD had a lower risk (RR = 0.95).

**Table 3.** Adjusted log-binomial model for UPC index by, gender, age, and centrality index for patients with asthma, diabetes mellitus, COPD, or heart failure, 2014.

Diagnoses	Asthma			COPD^a^			Diabetes mellitus			Heart failure		
	UPC^b^ ≥0.75(%)	RR	(95% CI)	UPC ≥0.75(%)	RR	(95% CI)	UPC ≥0.75(%)	RR	(95% CI)	UPC ≥0.75(%)	RR	(95% CI)
Gender												
Female	47.5	0.97	(0.93–1.01)	49.2	0.95	(0.91–0.98)*	58.7	1.0	(0.99–1.01)	72.4	1.13	(1.08–1.17)**
Male	42.3	ref.		51.5	ref.		58.6	ref.		62.1	ref.	
Age groups (years)												
≤18	28.6	0.51	(0.47–0.54)**	45.4	0.89	(0.47–1.69)	1.1	0.02	(0.01–0.4)**	50.0	0.89	(0.33–2.38)
19–44	42.9	0.76	(0.72–0.81)**	51.0	0.95	(0.78–1.16)	34.4	0.57	(0.55–0.59)**	49.1	0.87	(0.66–1.15)
45–54	50.4	0.90	(0.84–0.96)*	52.6	1.01	(0.93–1.10)	53.3	0.89	(0.87–0.90)**	45.4	0.81	(0.65–1.0)*
55–64	55.8	ref.		51.5	ref.		60.0	ref.		56.3	ref.	
65–74	58.8	1.04	(0.99–1.10)*	48.3	0.94	(0.90–0.99)*	62.2	1.04	(1.03–1.06)**	57.8	1.03	(0.93–1.15)
≥75	63.8	1.14	(1.08–1.21)**	51.8	1.01	(0.96–1.06)	66.8	1.12	(1.10–1.14)**	71.9	1.25	(1.14–1.37)**
Centrality index^c^												
1 (most urban)	46.7	ref.		52.6	ref.		62.3	ref.		70.9	ref.	
2	47.2	1.01	(0.96–1.07)	55.3	1.05	(1.0–1.11)	60.8	0.97	(0.96–0.99)*	69.8	1.01	(0.95–1.07)
3	45.9	0.97	(0.92–1.02)	51.7	0.98	(0.93–1.04)	59.6	0.95	(0.94–0.97)**	68.2	0.98	(0.92–1.04)
4	46.2	0.99	(0.93–1.05)	49.1	0.93	(0.88–0.99)*	57.0	0.91	(0.89–0.93)**	64.9	0.93	(0.87–1.0)*
5	31.7	0.68	(0.62–0.76)**	38.8	0.73	(0.67–0.80)**	51.6	0.82	(0.80–0.84)**	58.9	0.85	(0.78–0.93)*
6 (most rural)	32.2	0.66	(0.56–0.79)**	28.7	0.54	(0.47–0.63)**	42.0	0.66	(0.63–0.70)**	46.0	0.65	(0.54–0.79)**

The UPC index is dichotomized as UPC <0.74 and UPC ≥0.75 and proportion of patients with mean UPC ≥0.75 is also presented.

^a^COPD = chronic obstructive pulmonary disease.

^b^UPC = usual provider of care index.

^c^Centrality index 1 represents the most urban and 6 the most rural.

*P < 0.05.

**P < 0.001.

We found statistically significant increased RR for a high UPC by increasing age in patients with asthma and diabetes mellitus, while this was found only for the oldest age group (≥75) for heart failure. Living in rural areas gave a lower RR for a high UPC in all 4 populations.

In unadjusted model analyses, RR was statistically significant for high UPC by increasing educational level in patients with diabetes mellitus and heart failure (results not shown).

Table 4 presents the results from the adjusted log-binomial regression model for COCI and proportion of patients with COCI ≥0.75. Results from the adjusted log-binomial regression model for COCI were similar to UPC. Female with heart failure showed a higher risk of high COCI (RR = 16%) compared with male patients. In patients with asthma the probability of high COCI increased by increasing age.

**Table 4.** Adjusted log-binomial model for Bice–Boxerman continuity of care score (COCI) by gender, age, and centrality index for patients with asthma, diabetes mellitus, COPD, or heart failure, 2014.

Diagnoses	Asthma			COPD^a^			Diabetes mellitus			Heart failure		
	COCI^b^ ≥0.75(%)	RR	(95% CI)	COCI ≥0.75(%)	RR	(95% CI)	COCI ≥0.75(%)	RR	(95% CI)	COCI ≥0.75(%)	RR	(95% CI)
Gender												
Female	54.8	0.94	(0.91–0.98)*	43.7	0.94	(0.90–0.98)*	48.2	0.99	(0.97–1.0)	70.9	1.16	(1.11–1.22)**
Male	55.4	ref.		46.3	ref.		48.3	ref.		59.3	ref.	
Age groups (years)												
≤18	50.5	0.85	(0.81–0.90)**	72.7	1.64	(1.16–2.32)*	52.8	1.11	(1.03–1.20)*	100	1.00	(1.0–1.0)
19–44	51.0	0.87	(0.83–0.92)**	52.1	1.10	(0.91–1.34)	35.8	0.75	(0.72–0.78)**	61.4	1.18	(0.94–1.48)
45–54	54.5	0.94	(0.88–0.99)*	46.9	1.02	(0.93–1.13)	43.5	0.91	(0.88–0.93)**	51.3	0.98	(0.80–1.19)
55–64	58.0	ref.		45.6	ref.		47.9	ref.		53.0	ref.	
65–74	61.7	1.06	(1.0–1.12)*	43.6	0.96	(0.91–1.01)	48.5	1.02	(1.0–1.04)	56.0	1.07	(0.95–1.19)
≥75	66.5	1.15	(1.09–1.22)**	45.7	1.01	(0.95–1.06)	55.3	1.16	(1.14–1.19)**	69.1	1.28	(1.16–1.41)**
Centrality index^c^												
1 (most urban)	57.2	ref.		46.2	ref.		52.7	ref.		65.7	ref.	
2	57.4	1.01	(0.96–1.05)	49.4	1.07	(1.01–1.14)*	49.6	0.94	(0.92–0.96)**	67.2	1.05	(0.98–1.12)
3	55.7	0.98	(0.93–1.02)	46.5	1.01	(0.95–1.07)	47.3	0.89	(0.88–0.91)**	66.3	1.03	(0.96–1.10)
4	53.6	0.94	(0.90–0.99)*	43.1	0.93	(0.87–1.0)*	46.8	0.88	(0.86–0.90)**	62.2	0.96	(0.89–1.03)
5	43.9	0.77	(0.71–0.83)**	35.0	0.76	(0.69–0.83)**	44.6	0.83	(0.81–0.86)**	60.6	0.94	(0.86–1.03)
6 (most rural)	46.4	0.80	(0.70–0.92)*	29.4	0.63	(0.55–0.74)**	39.0	0.72	(0.68–0.76)**	50.4	0.77	(0.64–0.91)*

Bice–Boxerman continuity of care score is dichotomized as COCI <0.74 and COCI ≥0.75 and proportion of patients with mean COCI ≥0.75 is also presented.

^a^COPD = chronic obstructive pulmonary disease.

^b^COCI = Bice–Boxerman continuity of care score.

^c^Centrality index 1 represents the most urban and 6 the most rural.

*P < 0.05.

**P < 0.001.

## Discussion

In this study investigating CoC for asthma, diabetes mellitus, COPD, and heart failure we measured the highest UPC and COCI for patients with heart failure, particularly for female patients. UPC increased by increasing age for all the diagnoses, while COCI increased for patients with asthma and showed an inverse relationship with age for COPD. Both indices decreased significantly in rural areas.

### CoC across healthcare levels

We chose UPC as a measure of continuity with RGP, supplemented with COCI to determine total CoC across care levels. The rather high UPC shows that the majority of patients mainly consulted their RGP, indicating high RGP continuity, consistent with a previous study on the Norwegian patient list system.^20^ However, in some subgroups, COCI had a greater value than UPC, indicating higher CoC with other providers such as another GP or a specialist. We assume many of these patients have severe or complex conditions requiring follow-up by specialists. These cases highlight the importance of continuous shared care between primary and specialist care to manage such conditions. A Cochrane review from 2017 suggests that shared care between primary and specialist care improves outcomes for patients with depression but lacks sufficient evidence for its effect on other chronic conditions.^32^ This finding points out the limited research on multidisciplinary care and the need for further investigations.

### Gender

A previous study on continuity in primary care revealed generally higher CoC for female patients,^33^ but we do not have data in our study to replicate this result. Based on our results, only female patients with heart failure and asthma have higher CoC than their counterparts.

### Age groups

UPC increased by increasing age for all 4 diagnoses. High CoC with RGP is beneficial for older patients as they often have several chronic and complex conditions. Also, high RGP continuity is linked to reduced hospitalization and lower risk of mortality.^2,4^ Among patients with COPD, diabetes mellitus, and heart failure, COCI was the highest for patients ≤18 years old, in contrast to UPC which was the lowest in this age group. Specialists have a major role in managing these conditions in young patients, thus leading to higher CoC within specialist care. Additionally, decreasing COCI with age in patients with COPD might reflect increased utilization of OOH services.

### Urban versus rural

Both UPC and COCI were lower in rural areas indicating lower CoC with RGPs and involvement of several providers, respectively. We assume the main reason for lower CoC in rural areas is RGP scheme instability due to recruitment difficulties. This leads to increased employment of locum doctors which may contribute to increased utilization of OOH services. Distance to RGP services might also be a reason for fewer contacts. A Norwegian study found lower utilization rates for patients living far from OOH services,^34^ but we lack evidence for similar conclusion for RGPs. This highlights policymakers’ important task in providing a good RGP coverage in rural areas to maintain RGP continuity.

In our study, CoC decreased by increasing educational level in patients with diabetes mellitus and heart failure. This finding was unexpected and warrants further research.

Our findings point out the magnitude of CoC across care levels to provide a continuous and comprehensive patient care. We believe our results are transferrable to countries with similar healthcare system with patient list or GPs as gatekeepers.

### Strengths and limitations

A major strength of this study is acquisition of data from nationwide registries including the whole population and all consultations in different parts of healthcare system, thus limiting the possibility of selection bias. Nevertheless, our strict inclusion criterion of minimum 2 disease-related consultations during 2014 led to inclusion of rather small proportions of the total patient populations. From the source population, we estimate our study population for diabetes mellitus to be around one third and the other diagnoses around 10%–15%. This indicates that our study populations may represent seriously ill patients or those with frequent use of healthcare. However, this could not be investigated further since we do not have information on severity of their illnesses. Additionally, our inclusion criteria led to exclusion of those who had changed their RGP during 2014. Lack of CoC could be a reason for changing RGP. However, less than 5% of patients change their RGP each year, making this a factor without substantial effect.

Due to organizational matters, we could not identify physicians in Bergen OOH service during office hours and hospital outpatient clinics. Thus, each institution was calculated as 1 provider. When estimating COCI we assumed these patients consulted the same physician at hospital outpatient clinics, but there is a chance of meeting different physicians at each consultation. This would result in COCI overestimating CoC. Anyhow, these consultations and the ones from Bergen OOH service comprise a small proportion of total number of consultations. This study is limited to somatic conditions and our findings cannot be extrapolated to psychiatric disorders.

## Conclusions

Our findings suggest that CoC for the majority of patients with chronic diseases is high across the Norwegian healthcare system. Most patients mainly consult their RGP resulting in high RGP continuity. In some cases with low RGP continuity, patients nevertheless achieve high CoC with specialist care. High CoC for patients with chronic diseases is associated with older age and living in urban areas. Overall, these findings underline that policymakers face challenges in healthcare planning to maintain CoC for patients with chronic diseases.

## Data Availability

HELFO, NPR, and SSB provided the data. Due to restrictions by the Norwegian Data Protection Authority, data cannot be shared publicly.
